# The efficacy and safety of Chinese herbal compound combined with western medicine for amyotrophic lateral sclerosis: A protocol for systematic review and meta-analysis

**DOI:** 10.1097/MD.0000000000021933

**Published:** 2020-10-23

**Authors:** Lei Sun, Wenjuan Zhao, Mingliang Yan, Bin Yang, Peng Xiong, Shengjie Zhao

**Affiliations:** a Shaanxi University of Chinese Medicine, Xixian New Area; b Affiliated Hospital of Shaanxi University of Chinese Medicine, Xianyang; c Yan’an Municipal Hospital of Traditional Chinese Medicine, Yan’an, Shaanxi Province, China.

**Keywords:** amyotrophic lateral sclerosis, Chinese herbal compound, meta-analysis, protocol, systematic evaluation

## Abstract

**Background:**

Traditional Chinese medicine (TCM) compound formulations are selected according to different populations, with strong targeting and less adverse reactions. As a complex disease, amyotrophic lateral sclerosis (ALS) has limited efficacy in the use of conventional treatment regiments, short life cycle, high cost, many side effects, and low quality of life. It is urgent to seek new alternative therapies. Clinical practice shows that Chinese herbal compound combined with western medicine has certain therapeutic advantages, but there is no evidence of evidence-based medicine. The purpose of this study was to evaluate the efficacy and safety of Chinese herbal compound combined with western medicine in the treatment of ALS.

**Methods:**

Use computer to retrieve English database (PubMed, Embase, Web of Science, the Cochrane Library) and Chinese database (CNKI, Wanfang Database, Weipu database, and China Biomedical Literature Service System), moreover manually retrieve Baidu academic and Google academic from the establishment of the database to 2020 July for randomized controlled clinical study on ALS treated with compound Chinese medicine with western medicine therapy, 2 researchers independently conducted data extraction and literature quality evaluation on the quality of the included studies, and meta-analysis of the included literature was carried out using RevMan5.3 software.

**Results:**

This study evaluated the efficacy and safety of TCM combined with western medicine in the treatment of ALS by means of effective rate, improved Norris scale, ALS Functional Rating Scale, TCM syndrome score, and adverse reaction incidence.

**Conclusion:**

This study will provide reliable evidence for the clinical application of Chinese herbal compound combined with western medicine in the treatment of ALS.

OSF registration number: DOI 10.17605/OSF.IO/R5XG4

## 1 Introduction

Amyotrophic lateral sclerosis (ALS) is a disease characterized by a progressive degeneration of upper motor neurons (MNs) in the motor cortex and lower MNs in the brainstem and the spinal cord.^[1]^ Death of MNs in the motor cortex, brainstem, and spinal cord of the brain can cause autonomic dysfunction, manifested as muscle spasms, weakness, and atrophy, which in turn lead to paralysis.^[1–2]^ due to muscle paralysis, swallowing, speaking, and breathing difficulties, life-threatening, survival time is generally 3 to 5 years, respiratory failure is the main mode of death in patients within 3 years after diagnosis.^[3]^ About 90% of ALS are sporadic and 10% are hereditary in families.^[4]^ According to statistics, the annual incidence of ALS in Europe is 2 to 16/10,000,^[5]^ and the ages of ALS are mainly during adulthood (peak age of 58–63 years).^[6]^ Over 60 other molecules have been investigated as potential treatments for ALS, but the clinical effects are not good.^[7]^ Traditional Chinese medicine (TCM) is being more and more applied in the treatment of ALS, a large number of clinical data confirmed that the compound Chinese medicine have good effect to improve symptoms,^[8]^ treatment based on syndrome differentiation according to the different groups in the treatment of ALS have advantages of multiple targets, multiple ways, less side effects, and low cost, as same as against oxidative stress, excitatory amino acid toxicity, neuroinflammation, and calcium cytotoxicity, etc.^[9]^

Although there are multiple randomized controlled study results show that the compound Chinese medicine combined with western medicine can slow ALS disease development, improve the clinical symptoms, and have less adverse reaction,^[10–14]^ However, there are differences among clinical trials in terms of research scheme and efficacy evaluation, resulting in uneven research results and to some extent, affecting the promotion of this therapy. Therefore, this study plans to systematically evaluate the efficacy and safety of Chinese herbal compound combined with western medicine in the treatment of ALS, so as to provide a reliable reference basis for the clinical application of Chinese herbal compound combined with western medicine in the treatment of ALS.

## 2 Methods

### 2.1 Protocol register

This protocol of systematic review and meta-analysis has been drafted under the guidance of the Preferred Reporting Items for Systematic Reviews and Meta-Analyses. Moreover, it has been registered on open science framework on July 27, 2020 (Registration number: DOI 10.17605/OSF.IO/R5XG4).

### 2.2 Ethics

Since this is a protocol with no patient recruitment and personal information collection, the approval of the ethics committee is not required.

### 2.3 Eligibility criteria

#### 2.3.1 Types of studies

We will collect all available randomized controlled trails on Chinese herbal compound combined with western medicine in the treatment of ALS, regardless of blinding, publication status, region, but Language will be restricted to Chinese and English.

#### 2.3.2 Research objects

Patients with ALS are clearly diagnosed regardless of nationality, race, age, sex, or course of disease.

#### 2.3.3 Intervention measures

The treatment group was treated with Chinese herbal compound combined with Western medicine. The control group was treated with western medicine only. (There is no restriction on the types, dosage forms, dose, and course of treatment of Chinese herbal compound and Western medicine.)

#### 2.3.4 Outcome indicators

(1) Primary outcome: the overall effective rate;
(2) Secondary outcomes:
  1. Improved Norris scale score^[15]^;
  2. TCM syndrome score;
  3. ALS functional rating scale^[16]^;
  4. Incidence of adverse reactions.

### 2.4 Exclusion criteria

(1) Studies published repeatedly;
(2) Studies published as abstracts and unable to obtain data after contacting the author;
(3) Studies with incomplete data or obvious errors;
(4) Studies with high bias risk assessed by randomization or allocation concealment.^[17]^
(5) Studies with no relevant outcome indicators.

### 2.5 Retrieval strategy

“Chinese Medicine”, “Chinese herbal medicine”, “Chinese medicine”, “ALS” were searched in Chinese databases, including CNKI, Wanfang Data Knowledge Service Platform, VIP Information Chinese Journal Service Platform, and China Biomedical Database. English retrieves words such as “Chinese medicine”, “Chinese herbal compound”, “Amyotrophic lateral sclerosis”, and “ALS” were searched in English database, including PubMed, EMBASE, Web of Science, the Cochrane Library. In addition, manual search will be conducted on Baidu academic and Google academic. The retrieval time was from the date of establishment of the database to July 2020, and all domestic and foreign literatures on Chinese herbal compound combined with Western medicine for ALS were collected. Take PubMed as an example, and the retrieval strategy is shown in Table 1.

**Table 1:**
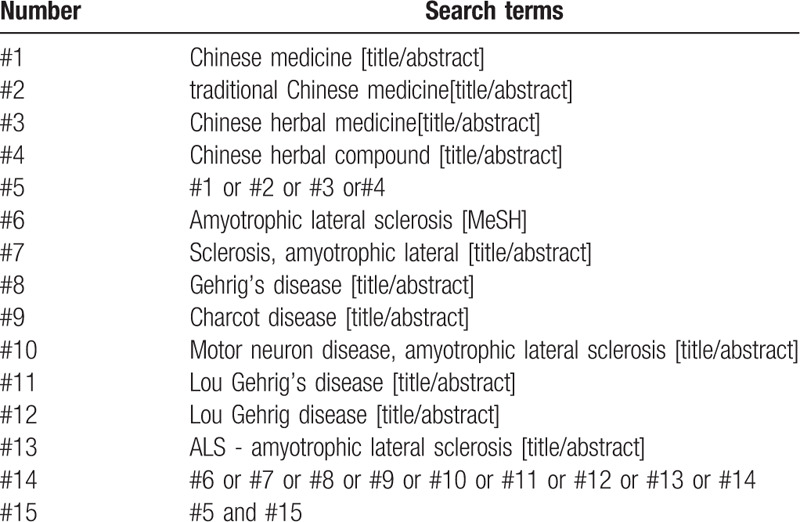
Search strategy in PubMed database.

### 2.6 Data screening and extraction

Referring to the method of research selection in version 5.0 of the Cochrane collaboration Network System Evaluator Manual, according to the Preferred Reporting Items for Systematic Reviews and Meta-Analyses flow chart, the 2 researchers used the EndNote X9 document management software to independently screen and check the literature according to the above inclusion and exclusion criteria, and check each other, if there were different opinions, negotiate with a third party to resolve the differences. At the same time, Excel 2013 was used to extract relevant information, including:

1. Clinical research (title, first author, publication date, sample size, sex ratio, average age, average course of disease);
2. Intervention measures (name, dose and course of treatment of western medicine in the control group; the compound name, TCM treatment, usage and dosage, course of treatment as well as the name and dosage of western medicine used in the treatment group);
3. Risk bias assessment factors in randomized controlled trials;
4. Observation index.

**Figure 1:**
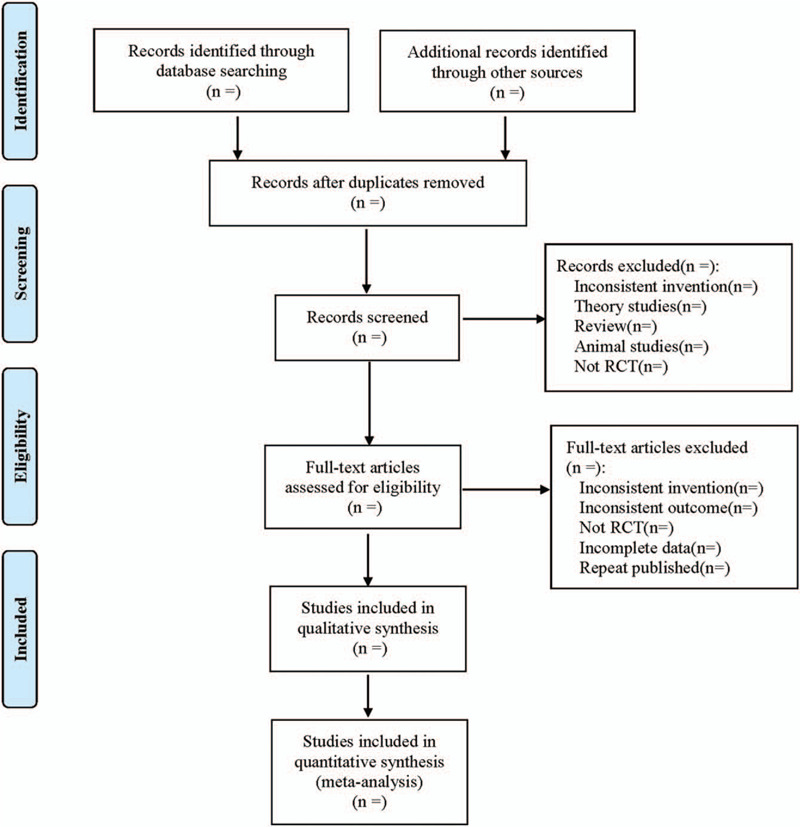
Flow diagram.

The literature selection process is shown in Figure 1.

### 2.7 Literature quality evaluation

Using the Cochrane collaboration's tool for assessing risk of bias to assess the risk of bias assessment included in the study. According to the performance of the included literature in the above evaluation items, the 2 researchers will give low risk, unclear and high risk judgments one by one, and cross-check after completion respectively. In case of differences, discussion will be conducted. If no agreement can be reached, discussion will be made with the researchers in the third party.

### 2.8 Statistical analysis

#### 2.8.1 Data analysis and processing

The RevMan 5.3 software was used for statistical analysis. For dichotomous variables, relative risk was used for statistics. For continuous outcomes, weighted mean difference was selected when the tools and units of measurement indicators are the same, standardized mean difference was selected when the tools and units of measurement indicators are different, and all the above were represented by effect value and 95% confidence interval. The heterogeneity was determined by χ^2^ and I^2^ values. if (*P* ≥ . 1, I^2^≤50%) indicated low heterogeneity, fixed effect model was used for meta-analysis. If (*P *< . 1, I^2^ > 50%)indicated heterogeneity among studies, and the source of heterogeneity would be explored through subgroup analysis. If there was no obvious clinical or methodological heterogeneity, it would be considered as statistical heterogeneity, and the random-effect model would be used for analysis. Descriptive analysis was used instead of meta-analysis if there was significant clinical heterogeneity between the 2 groups and subgroup analysis was not available.

#### 2.8.2 Dealing with missing data

If there is missing data in the article, contact the author via email for additional information. If the author cannot be contacted, or the author has lost relevant data, descriptive analysis will be conducted instead of meta-analysis.

#### 2.8.3 Subgroup analysis

According to the age of the patients, ALS can be divided into young and old for subgroup analysis. Subgroup analysis was performed according to the course of treatment. Subgroup analysis was carried out according to different TCM compound treatments in the treatment group.

#### 2.8.4 Sensitivity analysis

In order to determine the stability of outcome indicators, sensitivity analysis was used to analyze each outcome indicator.

#### 2.8.5 Assessment of reporting biases

Funnel plots were used to assess publication bias if no fewer than 10 studies were included in an outcome measure. Moreover, Egger and Begg test were used for the evaluation of potential publication bias.

#### 2.8.6 Evidence quality evaluation

The Grading of Recommendations Assessment, Development, and Evaluation will be used to assess the quality of evidence. It contains 5 domains (bias risk, consistency, directness, precision, and publication bias). And the quality of evidence will be rated as high, moderate, low, and very low.

## 3 Discussion

The pathogenesis of ALS is not yet clear and may be related to excitatory amino acid toxicity, oxidative stress injury, mitochondrial abnormalities, gene mutations, protein instability, and neurotrophic factor deficiency, etc. Currently, there is no effective treatment that can cure or prevent the development of the disease. Riluzole is an inhibitor of glutamate neurotransmitter, the only drug approved by the US Food and Drug Administration in the early treatment of ALS. It has certain benefits on survival rate, but only prolongs life expectancy by 2 to 4 months.^[18]^ It is expensive and has side effects such as nausea, fatigue, and elevated liver enzyme level.^[19–20]^ Edaravone is another drug on the market in Japan that has been considered effective through phase III clinical verification. The exact cellular and molecular targets are still unknown, but it is also expensive and has many adverse reactions.^[21]^ So there is an urgent need to find new alternative therapies.

ALS belongs to the category of “flaccidity disease” (weizheng) in TCM. Due to insufficient endowment, deficiency of viscera and organs, and loss of nourishment of muscle channels,^[22]^ ALS is manifested as lean muscle, impotence and weakness. According to the syndrome differentiation and classification of TCM, ALS can be divided into: syndrome of heat and fluid injury of lung, syndrome of dampness-heat infiltration, syndrome of weakness of spleen and stomach, syndrome of deficiency of liver and kidney, and syndrome of vein stasis.^[23]^ According to the differentiation of syndromes in different populations, TCM combined with western medicine can significantly improve the curative effect. Su Guoliang et al^[12]^ took Yiqi Qiangji decoction combined with riluzole as the treatment group and with riluzole alone in control group to treat ALS for 3 months, and the results showed that the treatment group was superior to the control group in terms of clinical symptoms and TCM syndrome scores. Pan Weidong et al^[13]^ compared riluzole alone with modified Sijunzi decoction combined with western medicine, and the results showed that Chinese herbal compound combined with western medicine had more advantages than western medicine alone in delaying the development of ALS, with fewer side effects and lower price. Therefore, the systematic evaluation of the efficacy and safety of Chinese herbal compound combined with western medicine in the treatment of ALS provides a reliable basis for this new alternative therapy.

However, this systematic review has some limitations. Due to the particularity of TCM decoction itself, the composition and dosage of prescriptions used in each study were different, which had certain influence on the combined results of outcome indexes. At the same time, due to the limitation of language ability, we only search literature in English and Chinese, and may ignore research or reports in other languages.

## Author contributions

**Data collection:** Mingliang Yan and Bin Yang

**Funding to support:** Shengjie Zhao.

**Literature search:** Lei Sun and Wenjuan Zhao

**Software operation:** Peng Xiong.

**Supervision:** Shengjie Zhao.

**Writing – original draft:** Lei Sun and Wenjuan Zhao.

**Writing – review & editing:** Lei Sun and Shengjie Zhao.
